# Supplementary material for: Meta-QTL Analysis and Identification of Candidate Genes Associated with Stalk Lodging in Maize (Zea mays L.)
Source: Curr Issues Mol Biol. 2025 Sep 23;47(10):792. doi: 10.3390/cimb47100792 (PMC12562863; doi:10.3390/cimb47100792)
Supplement: Supplementary file 1 [file cimb-47-00792-s001.zip › Supplementrary data/Figure S1.pdf]

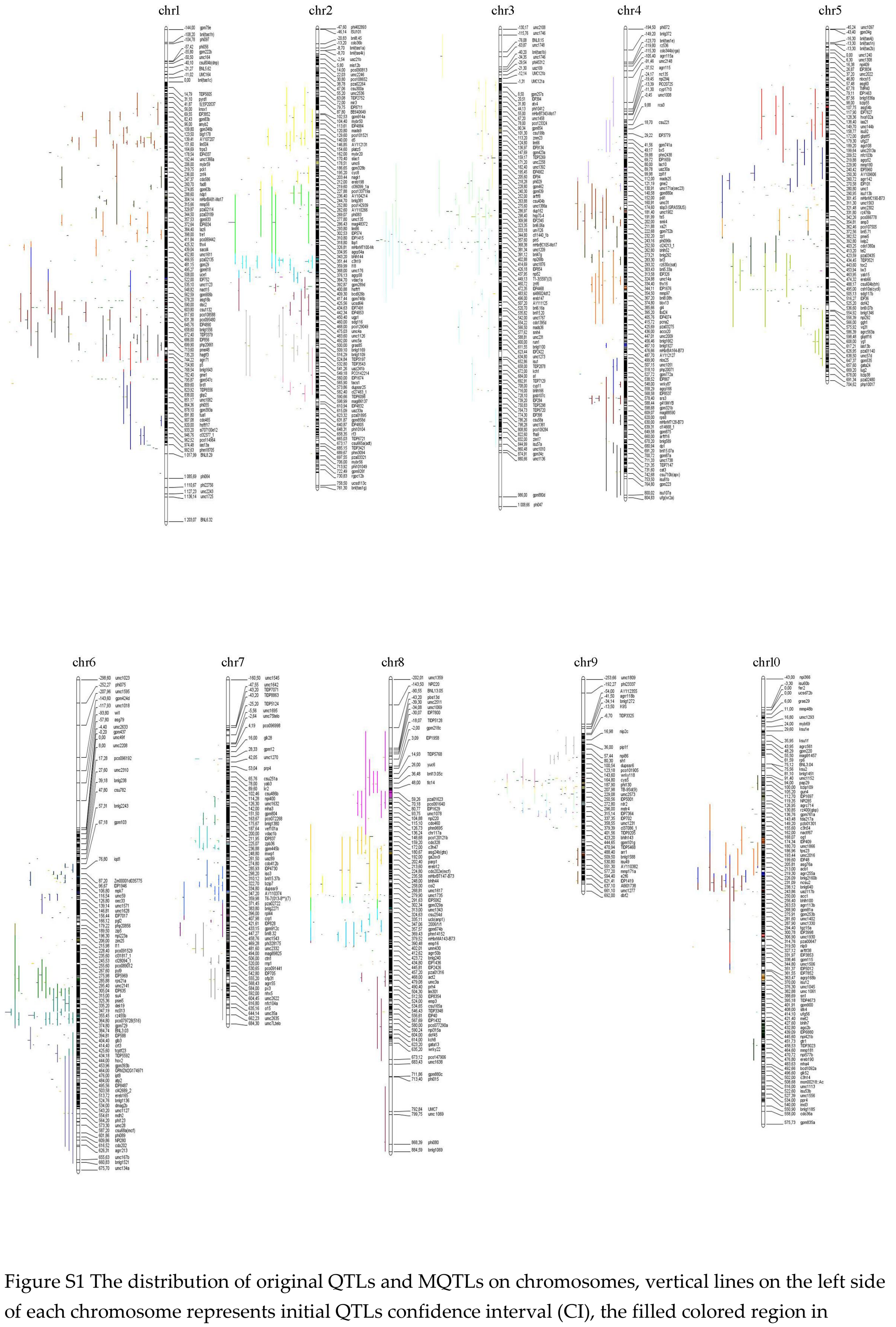

Figure S1 The distribution of original QTLs and MQTLs on chromosomes, vertical lines on the left side of each chromosome represents initial QTLs confidence interval (CI), the filled colored region in chromosome represents the identified MQTLs.
